# Nominal logistic regression analysis of variables determining needle visibility in ultrasound images – a full factorial cadaver study

**DOI:** 10.1186/s12871-023-02339-y

**Published:** 2023-11-10

**Authors:** Hanns-Christian Dinges, Julia Hoeft, Valér Michael Cornelius, Thorsten Steinfeldt, Thomas Wiesmann, Hinnerk Wulf, Ann-Kristin Schubert

**Affiliations:** 1 Department of Anesthesiology and Intensive Care Medicine, University Hospital Marburg, Philipps University of Marburg, 35033 Baldingerstraße, Marburg, Germany; 2 Department of Anesthesiology, Intensive Care and Pain Medicine, BG Unfallklinik Frankfurt am Main, Frankfurt am Main, Germany; 3 Department of Anesthesiology and Intensive Care Medicine, Diakoneo Diak Klinikum Schwäbisch-Hall, Schwäbisch- Hall, Germany

**Keywords:** Needle visibility, Ultrasound-guided regional anesthesia, Echogenic needle, Nominal logistic regression

## Abstract

**Background:**

Needle visualization is essential to avoid vascular puncture and nerve injury in ultrasound-guided regional anesthesia. Several factors that statistically influence needle visibility have been described but the dimensions of their individual impact remain unclear. This study aimed to quantify the impact of various independent factors on ultrasound needle visibility.

**Methods:**

A total of 1500 ultrasound videos of in-plane needle insertions were obtained in embalmed cadavers with ten different commercially available echogenic and non-echogenic needles at different insertion angles and bevel orientations in a full factorial study design. The visibility of needle tip and shaft were rated as “good” or “poor” visibility. Nominal logistic regression analyses were calculated for the visibility of the needle tip and shaft.

**Results:**

SonoPlex Stim Sprotte, SonoTAP Facet (needle tip and shaft) and Spinostar PencilPoint (needle tip)), insertion angle and bevel orientation were associated with good ultrasound visibility, reaching statistical significance (p < 0.05). The range of the effect on the log-odds scale for needle tip visibility was largest for the insertion angle with 6.33, followed by the tissue condition (3.76), bevel orientation (1.45) and the needle types (1.25). Regarding the needle shaft visibility, the largest effect range was observed with the insertion angle (7.36), followed by the tissue conditions with 3.96, needle type (1.86) and bevel orientation (0.95).

**Conclusion:**

In-plane needle visibility in ultrasound images depends mainly on the insertion angle, as expected. This is closely followed by the tissue condition, which is a factor related to the patient, thus cannot be altered to improve needle visibility. In the dimensions of the log-odds scale, the choice of a specific needle is far less important towards achieving a good visualization, whereas optimizing the bevel orientation can have a larger impact than the needle choice. Concluding from the relative dimensions of factors that determine needle visibility in this model, the importance of needles with echogenic features may be overrated.

**Supplementary Information:**

The online version contains supplementary material available at 10.1186/s12871-023-02339-y.

## Key message

**What is already known on this topic** – A plethora of factors that determine needle visibility in ultrasound images have been described statistically, including echogenicity of commercially available needles.

**What this study adds** – This study gives a precise quantification of how these factors impact the needle visibility individually and describes their limits.

**How this study might affect research, practice or policy** – The results of this study can help physicians performing regional anesthesia to make better choices to improve their needle visibility for safer needle guidance.

**Supplementary Information:**

The online version contains supplementary material available at 10.1186/s12871-023-02339-y.

## Introduction

Needle visualization is essential for the successful and safe use of ultrasound-guided regional anesthesia. A clear visualization especially of the needle tip is important to avoid unintended adverse events as accidental vascular puncture, nerve injury or intraneural injection [1, 2].

There are many factors that determine needle visibility in ultrasound imaging. Some of them are related to the material and technical equipment as image quality and resolution of the ultrasound or technical improvements in ultrasound techniques as spatial compound imaging [3] or optical reflexional spectrophotometry [4, 5]. Echogenic needles are designed to improve needle visibility especially when in-plane-techniques are used. They use surface modifications such as special polymer coatings, texturing of the needle or etchings on the shaft or wire guides [6] which cause higher ultrasound reflection when compared to conventional needles with smooth surfaces [7].

Other factors are more related to the physique of the patient such as depth of the targeted structures and the determined insertion angle.

Lastly, the practitioner skills to position and angulate the needle and the ultrasound probe in relation to each other are a major factor for successful and safe needle guidance.

While these factors are all known, their individual impact on the needle visualization is still controversially debated. The objective of the present study was to quantify the effect of the explanatory factors: insertion angle, needle type, bevel orientation and tissue on in-plane needle visibility.

## Methods

### Study design

This study was designed as a full factorial experiment. The factors of the design were the insertion angle with 5 levels (10, 20, 30, 40 and 45 degree), the bevel orientation with 3 levels (up, down, sideways), the needle type with 10 distinct commercially available needle models that were dichotomized into echogenic or non-echogenic surface structure. The study was performed in a cadaver model using 10 embalmed cadavers, therefore the cadaver itself was considered a level of the explanatory factor “tissue constitution”.

### Ethical approval

The embalmed cadavers (four females, six males, aged 64–98 years) were provided by the Department of Anatomy of Philipps University Marburg according to the department’s ethical guidelines for the use of human cadavers. All body donors had intended to donate their body to medical science and education in their testamentary disposition.

### Insertion technique and ultrasound image recording

Randomization of the testing order was achieved by number generating software (www.randomizer.org, 2000 sets of 3 unique numbers per set).

Needle insertion technique was performed in a standardized manner at the ventral thigh of the cadavers. Five different insertion angles (10°, 20°, 30°, 40° and 45°) determined between linear probe and needle shaft according to Schafhalter-Zoppoth et al. [6] were analyzed. Consistency in insertion angle and use of in-plane approach was assured by a static needle guide (Infiniti Plus; Civco, Kalona, Iowa) attached to the probe. Three different orientations of bevel (up, down, side) were assessed for each needle at each angle, resulting in a total number of 1500 video clips. Bevel up was defined as bevel orientated to face the transducer.

Ultrasonography was performed under standard conditions using conventional B-Mode imaging, depth 2.7 cm. The investigator performing the needle insertion and ultrasound image recording was not blinded. A linear transducer was applied (HFL38x; frequency range 6–13 MHz; SonoSite, Bothell, Washington). Needle insertion and withdrawal were visualized and recorded in video clips using the ultrasound machine SonoSite S-Nerve including compound imaging technology (SonoSite, Bothell, Washington).

### Characteristics of investigated needles

Ten conventionally available different needle types were compared in this study. All needles had a 22-G diameter and a length of 70–90 millimeters depending on availability. Characteristics of investigated needles are shown in Table 1.

**Table 1:** Characteristics of tested needles

Number	Manufacturer	Name	Tip Design	Echogenicity	Diameter (Gauge)	Length (mm)
1	Pajunk	Sonoplex Stim	Facet	echogenic	22 G	80 mm
2	Pajunk	Sonoplex Stim	Sprotte	echogenic	22 G	90 mm
3	Pajunk	SonoTAP	Facet	echogenic	22 G	80 mm
4	Teleflex	SpinoStar Ballpen	Ballpen	non-echogenic	22 G	90 mm
5	Teleflex	Spinostar Pencil-Point OPTI	Sprotte	non-echogenic	22 G	90 mm
6	Teleflex	SpinoStar Standard	Facet	non-echogenic	22 G	90 mm
7	B. Braun	Stimuplex D	Facet 15°	non-echogenic	22 G	80 mm
8	B. Braun	Stimuplex D	Facet 30°	non-echogenic	22 G	80 mm
9	B.Braun	Stimuplex Ultra 360	Facet 30°	echogenic	22 G	80 mm
10	Teleflex	UltraQuik PNB	Facet	echogenic	22 G	70 mm

Characteristics of tested needles. Manufacturers: B. Braun, Melsungen, Germany; Pajunk, Geisingen, Germany; Teleflex, Wayne, Pennsylvania, USA

### Ultrasound image assessment

All 1500 video clips were processed, editing the duration of video sequence and removal of any kind of text to assure blinding, before evaluation. The assessment of the recorded video clips was performed by an independent anesthesiologist experienced in ultrasound-guided regional anesthesia and not involved in the acquisition of the ultrasound video sequences.

**Fig. 1:**
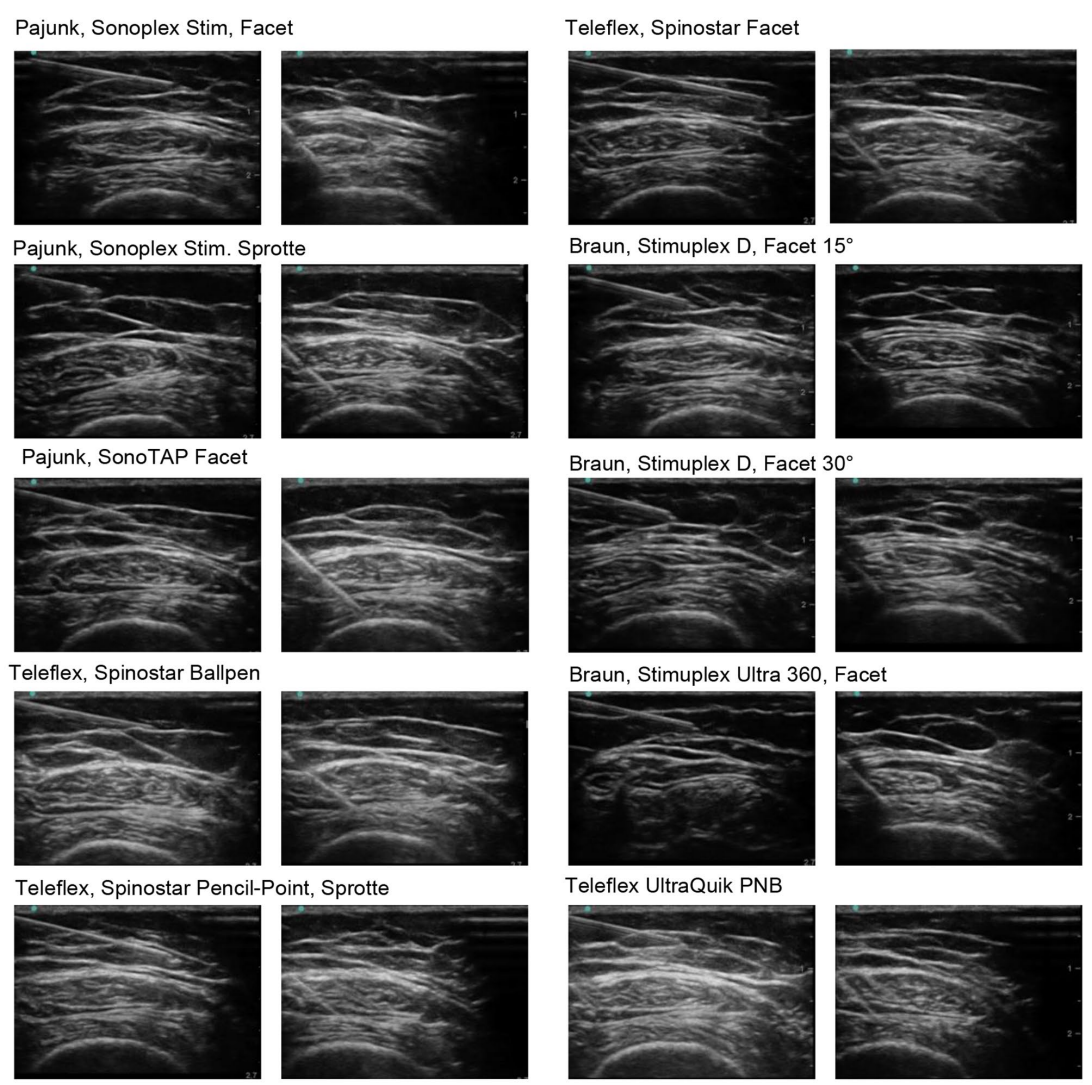
Examples of ultrasound images for all needles for insertion angle 10° (first and third column) and 45° (second and fourth column)

The visibility of needle tip and shaft were rated as “good” or “poor” visibility. Examples of ultrasound images for all needles are presented in Fig. 1.

### Data record

The final data set included 1500 judgements for the visibility of the needle tip and shaft respectively.

### Nominal logistic regression

This analysis combined all variables (categorical and continuous) that were collected in the full factorial design of this study. Two regression analyses were calculated. The first dependent variable was the visibility of the needle tip, the second one was the visibility of the needle shaft. The dependent variables had two possible expressions (“good” or “poor”).

Independent variables were the 10 different needles, the 10 different tissues (embalmed cadavers), the 3 bevel orientations (up, down, side), and the insertion angle as the only interval scaled independent variable (10–45 degree of angulation).

### Statistical assessment

All regression analyses were done with JMP® Pro Version 16.0.0. All other statistical analysis was performed using Excel (Microsoft Excel for Mac, Version 16.24, Redmond, Washington).

Statistical significance was defined at p < 0.05. Goodness of fit is reported as the generalized R^2^ proposed by Nagelkerke et al. [8].

## Results

### Needle tip visibility

Categorical variables in this analysis are reported in comparison to reference variables. These references were the cadaver 10 for the tissue, the UltraQuik PNB for the needle type and the upwards position for the bevel orientation. The results of the nominal logistic regression for the visibility of the needle tip are presented in Table 2. In comparison with the reference categories, the probability of a good visibility was decreased with cadavers 1–3, while it was significantly increased with cadavers 6–9. For the needles, the SonoPlex Stim Facet, SonoPlex Stim Sprotte and SpinoStar PencilPoint were associated with a significantly higher probability of a good visibility, whereas the StimuPlex D 30° significantly decreased the probability. Regarding the bevel orientation, the bevel down increased, while the bevel side orientation decreased the visibility, both statistically significant compared to the reference bevel upwards orientation (all p < 0.05). The explanatory value of this regression analysis regarding the observed variance was 0.64 (generalized R^2^) indicating a good fit.

**Fig. 2:**
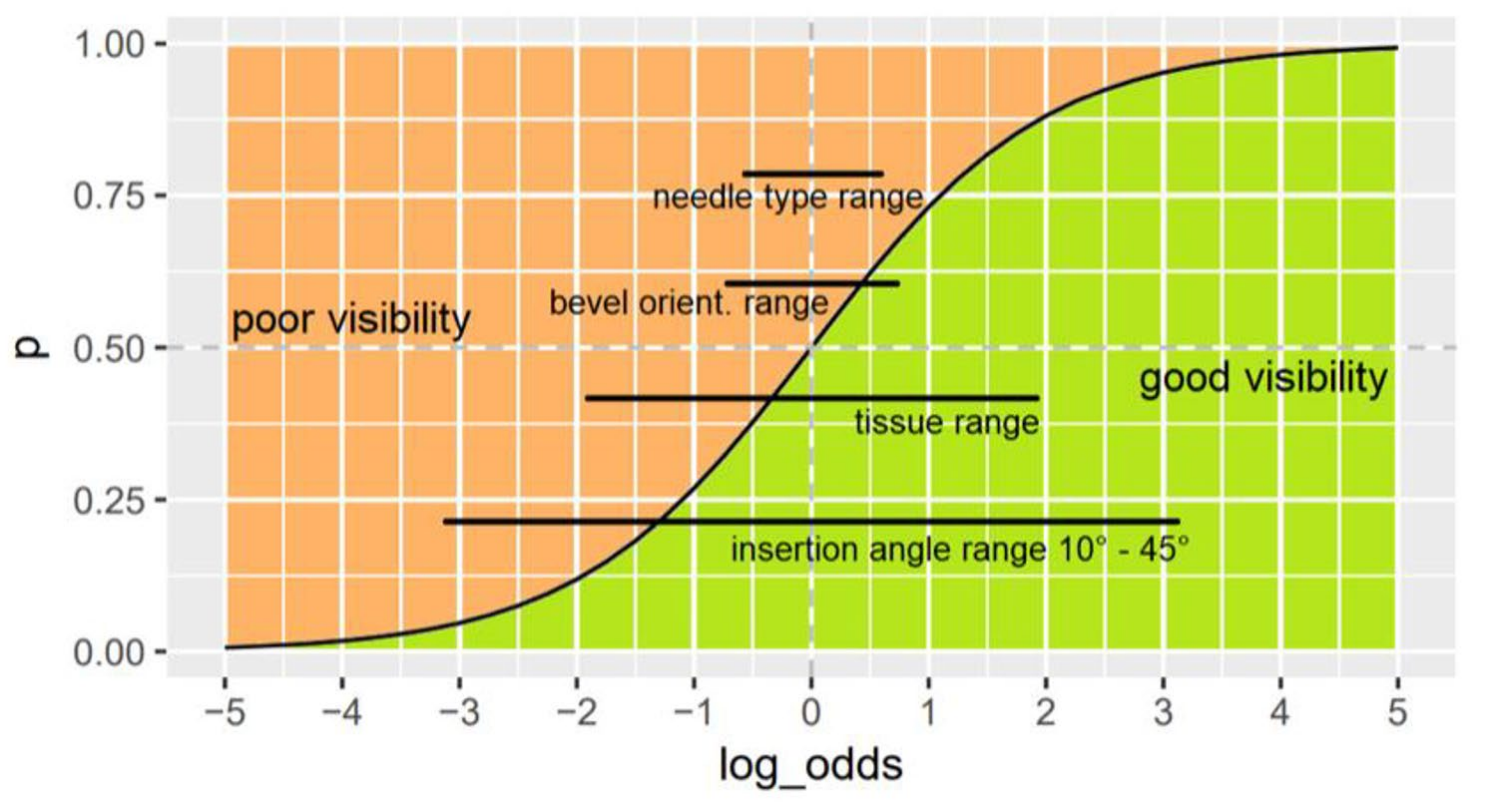
Probability (p) versus log-odds curve in relation to the total ranges of the factors insertion angle, tissue, bevel orientation and needle type on the visibility of the needle tip

The range in log-odds of the independent variables and their impact on the probability in relation to the effects they have on these scales are presented in Fig. 2. The largest was the range of the angulation when inserting a needle (10° for shallowest and 45° for steepest angle), which was 6.33 on the log-odds scale. This value is derived from the coefficient of -0.18 (95% CI -0.20 to -0.16) per degree, multiplied with the total range of degrees which was 35° (10 to 45). The range in tissue conditions, regarded as the difference between the estimates of cadavers with the highest, respectively lowest probability of a good needle tip visibility, was 3.76. According to this but smaller, were the ranges for bevel orientation with 1.45 and the needle types with 1.25. The intercept was 3.73 (95% CI 3.29 to 4.19).

**Table 2:** Nominal logistic regression for needle tip visibility

Term	Estimate	Std Error	ChiSquare	Prob > ChiSq	Lower 95%	Upper 95%
Intercept	3.73	0.23	266.56	< 0.0001*	3.29	4.19
tissue[cadaver 1]	-2.02	0.29	48.73	< 0.0001*	-2.60	-1.47
tissue[cadaver 2]	-0.79	0.25	10.04	0.0015*	-1.29	-0.31
tissue[cadaver 3]	-1.68	0.28	37.23	< 0.0001*	-2.23	-1.15
tissue[cadaver 4]	-0.01	0.24	0.00	0.98	-0.48	0.46
tissue[cadaver 5]	-0.01	0.24	0.00	0.98	-0.48	0.46
tissue[cadaver 6]	0.74	0.24	9.69	0.0019*	0.27	1.20
tissue[cadaver 7]	0.98	0.24	16.99	< 0.0001*	0.51	1.44
tissue[cadaver 8]	1.74	0.24	51.55	< 0.0001*	1.27	2.22
tissue[cadaver 9]	0.68	0.24	8.16	0.0043*	0.21	1.14
needle[SonoPlex Stim Facet]	0.14	0.24	0.33	0.57	-0.34	0.61
needle[SonoPlex Stim Sprotte]	0.58	0.24	5.93	0.0149*	0.11	1.05
needle[SonoTAP Facet]	0.58	0.24	5.93	0.0149*	0.11	1.05
needle[SpinoStar BallPen]	-0.46	0.25	3.39	0.07	-0.95	0.02
needle[SpinoStar PencilPoint]	0.52	0.24	4.72	0.0299*	0.05	0.99
needle[SpinoStar Standard Facet]	0.07	0.24	0.09	0.76	-0.40	0.55
needle[StimuPlex D 15°]	-0.12	0.24	0.25	0.62	-0.60	0.35
needle[StimuPlex D 30°]	-0.67	0.25	6.97	0.0083*	-1.17	-0.18
needle[StimuPlex Ultra]	-0.19	0.24	0.59	0.44	-0.67	0.29
bevel orientation[down]	0.84	0.12	50.25	< 0.0001*	0.61	1.07
bevel orientation[side]	-0.61	0.12	26.42	< 0.0001*	-0.85	-0.38
insertion angle (degree)	-0.18	0.01	367.89	< 0.0001*	-0.20	-0.16

Nominal logistic regression for needle tip visibility. Confidence limits are likelihood-based. For log odds of good/poor. Statistically significant results are asterisked

### Needle shaft visibility

The results of the regression for the visibility of the needle shaft are presented in Table 3. The reference variables for categorical variables are according to the needle tip visibility cadaver 10 for tissue, UltraQuik PNB for needle and “bevel up” for the needle orientation. The estimations of this regression analysis correlated well with the needle tip visibility results. The SpinoStar BallPen needle was associated with a statistically significant decrease in the probability of a good visibility, while the SpinoStar PencilPoint did not. Apart from these two needles, the results with statistical significance were the same as in the regression analysis for the needle tip visibility. The explanatory potential of this regression analysis was higher than for the needle tip regression analysis with a generalized R^2^ of 0.71. Regarding the ranges, the largest was still the insertion angle with 7.36 (-0.21 per degree, 95% CI -0.23 to -0.19), followed by the tissue conditions with 3.96. The order for the range of the needle type and bevel orientation changed with 1.86 (needle type) and 0.95 (bevel orientation). The intercept was 5.48 (95% CI 4.92 to 6.09).

**Table 3:** Nominal logistic regression for needle shaft visibility

Term	Estimate	Std Error	ChiSquare	Prob > ChiSq	Lower 95%	Upper 95%
Intercept	5.48	0.30	340.24	< 0.0001*	4.92	6.09
tissue[cadaver 1]	-2.47	0.29	73.12	< 0.0001*	-3.05	-1.92
tissue[cadaver 2]	-0.55	0.25	4.63	0.0314*	-1.05	-0.05
tissue[cadaver 3]	-2.39	0.29	69.53	< 0.0001*	-2.97	-1.84
tissue[cadaver 4]	0.42	0.25	2.91	0.09	-0.06	0.91
tissue[cadaver 5]	0.15	0.25	0.36	0.55	-0.34	0.64
tissue[cadaver 6]	1.22	0.25	23.91	< 0.0001*	0.74	1.72
tissue[cadaver 7]	1.36	0.25	29.09	< 0.0001*	0.87	1.85
tissue[cadaver 8]	1.49	0.25	34.67	< 0.0001*	1.00	1.99
tissue[cadaver 9]	0.83	0.25	11.07	0.0009*	0.34	1.32
needle[SonoPlex Stim Facet]	0.26	0.25	1.07	0.30	-0.23	0.74
needle[SonoPlex Stim Sprotte]	1.13	0.25	20.48	< 0.0001*	0.64	1.62
needle[SonoTAP Facet]	0.86	0.25	12.11	0.0005*	0.38	1.35
needle[SpinoStar BallPen]	-0.73	0.26	8.00	0.0047*	-1.24	-0.23
needle[SpinoStar PencilPoint]	-0.23	0.25	0.82	0.37	-0.73	0.26
needle[SpinoStar Standard Facet]	0.05	0.25	0.04	0.84	-0.44	0.54
needle[StimuPlex D 15°]	-0.44	0.25	3.01	0.08	-0.94	0.05
needle[StimuPlex D 30°]	-0.58	0.26	5.21	0.0224*	-1.09	-0.09
needle[StimuPlex Ultra]	0.19	0.25	0.58	0.45	-0.30	0.68
bevel orientation[down]	0.71	0.12	34.11	< 0.0001*	0.47	0.95
bevel orientation[side]	-0.24	0.12	3.99	0.0457*	-0.47	-0.01
insertion angle (degree)	-0.21	0.01	394.72	< 0.0001*	-0.23	-0.19

Nominal logistic regression for needle shaft visibility. Confidence limits are likelihood-based. For log odds of good/poor. Statistically significant results are asterisked

## Discussion

### Main findings

In a full factorial study design, we investigated the influence of insertion angle, needle type, bevel orientation and tissue on needle visibility in sonographic image sequences.

Our findings indicate that the SonoPlex Stim Sprotte, SonoTAP Facet and SpinoStar PencilPoint were the needles with best ultrasound visibility and that the bevel down orientation was associated with the best results all statistically significant. Furthermore, an increase of the insertion angle decreased the probability of good needle visibility, also with statistical significance. The tissue resembled by the different cadavers, has significant influence on the needle visibility as well.

Since the reference variables for groups of categorical variables were not necessarily the best or the worst in their category and because the estimate for angulation is given per degree, it is important to compare the total effect range of every group of variables. This allows to interpret by how much each measure impacts the needle visibility overall. Therefore, the effect ranges (from minimal to maximal) are presented on the log-odds scale in relation to the curve for probability in Fig. 2. The figure shows that the angulation affected needle tip and shaft visibility with a range of 6.33, respectively 7.36 on the log-odds scale. This resembles a large difference in the probability of good or poor visibility practically anywhere on the curve. The tissue conditions affected the probability of good or poor needle visibility with a smaller range of 3.76 and 3.96. The needle type and bevel orientation however, had a surprisingly small overall effect on the probability with ranges of 1.25 and 1.86 (needle type tip and shaft) and 1.45 and 0.95 (bevel orientation tip and shaft).

This indicates that despite certain echogenic needles may improve visibility with statistical significance compared to other needles, the effect of good angulation is still a multiple of that effect which can be expected from switching to a better, most likely more expensive needle. The tissue is a factor that cannot be controlled for in the clinical setting, but it also affects the needle visibility much more than the choice of a needle does. While the importance of limiting needle angulation for better ultrasound visibility is common knowledge, this relationship has not been quantified before and the effect of the needle type on needle visibility was much smaller than expected.

### The effect of echogenicity at steeper insertion angles

In the scientific literature echogenic needles have shown to improve the ultrasound visibility with statistical significance, especially at steeper insertion angles.

For better comparability with other studies on this topic, we have presented bar plots of our data, showing the visibility of needle tip and shaft of echogenic and non-echogenic needles in the supplemental content (Supplemental Fig. 1). Despite statistical significance at higher angles (Chi-Square-Test), the proportion of poor visibility remained high. At angles of 40–45° needle tip visibility was poor in 89–95% with echogenic needles and in 99–100% with non-echogenic needles. Needle shaft visibility was poor in 71–81% with echogenic needles and in 99–100% with non-echogenic needles. On other words, this would mean that non-echogenic needles are expected to almost never show good visibility at 40–45°, whereas 10–25% of echogenic needles are expected to have good visibility at these angles. In our opinion, this effect is smaller than expected, but can be of relevance in some cases, if it translates to clinical practice.

### Previous studies

Studies that investigated the visibility of tip or shaft of echogenic needles compared to non-echogenic ones regularly show a significant benefit of the echogenicity, especially at higher insertion angles of 30–75°. [1, 3, 9] This statistical significance is also present in our data. Unfortunately, these studies did not relate this effect to other measures like the actual effect of angulation or tissue.

The systematic review of Hovgesen et al. is the largest review on the topic and the advantages of echogenicity have been shown to be most apparent when using steeper insertion angles in relation to the transducer. [10] However, a quantification of needle visibility via scoring could not be done from the inhomogeneous reporting of the individual studies.

Guo et al. assessed needle visibility scores in a human Thiel Cadaver [1]. Primary endpoint in this study was needle visibility assessed by two independent reviewers.

Although, needle visibility could be improved using echogenic needles, in-plane-technique and spatial compound imaging, a high percentage of needles had shown only poor visibility [1, 3].

Maecken et al. did an investigation with visibility at 0- versus 45-degree angles in the animal model and concluded that visibility was severely limited at 45 degrees and that only few of the investigated echogenic needles had an “acceptable” visibility at that angle. [11].

To our knowledge, the relationship between angulation, tissue, bevel orientation and choice of an (echogenic) needle has not been quantified as precisely as we have done in this analysis.

### Limitations

This study was performed in a non-clinical setting using embalmed human cadavers. Needle visibility in embalmed cadavers is not identical, yet comparable with that in human. Other models (meat, artificial gel) are more problematic as they provide significantly higher needle visibility [12]. Therefore, this is only a minor limitation.

The visibility was subjectively rated by a single observer, which however, resembles clinical routine in most cases.

Taking the independent variable “tissue”, represented by the different cadavers, into the regression analysis is debatable, since in clinical practice, the patient itself cannot be altered. The estimates for the different cadavers have therefore no direct implications on how to improve needle visibility in general, but our aim was to present a statistical model with high explanatory value, which we achieved by taking the tissue into the analysis. Not considering it or not knowing about the impact of the tissue condition may also lead to frustration when trying to optimize visibility via controllable factors or to overambitious attempts to perform blocks in these situations. Having this knowledge on the other hand may take pressure off clinicians who abide from undertaking risky blocks under poor conditions. These aspects however are hypotheses, that are derived from the presented statistical model from data of embalmed cadavers and conclusions may not necessarily translate into the clinical setting.

## Conclusion

Needle visibility mostly depends on a shallow insertion angle, as expected, closely followed by the uncontrollable variable “tissue”. In the dimensions of the log-odds scale, the choice of a specific needle is far less important towards achieving a good visualization, whereas optimizing the bevel orientation of the needle can have a larger impact than manufactured echogenic features.

Concluding from the relative dimensions of factors that determine needle visibility in this model, the importance of needles with echogenic features may be overrated in the scientific debate, whereas the uncontrollable factor of the tissue or patient may be underestimated and might be mistaken for lack of personal skill when visibility conditions cannot be optimized in clinical practice.

To our knowledge, this study is the first to demonstrate these relationships and rank the factors in order of clinical relevance.

### Electronic supplementary material

Below is the link to the electronic supplementary material.

Supplementary Material 1

## Data Availability

The datasets used and/or analyzed during the current study are available from the corresponding author on reasonable request.
