# Supplementary material for: Nominal logistic regression analysis of variables determining needle visibility in ultrasound images – a full factorial cadaver study
Source: BMC Anesthesiol. 2023 Nov 10;23:369. doi: 10.1186/s12871-023-02339-y (PMC10636954; doi:10.1186/s12871-023-02339-y)
Supplement: Supplementary file 1 — Supplementary Material 1 [file 12871_2023_2339_MOESM1_ESM.pdf]

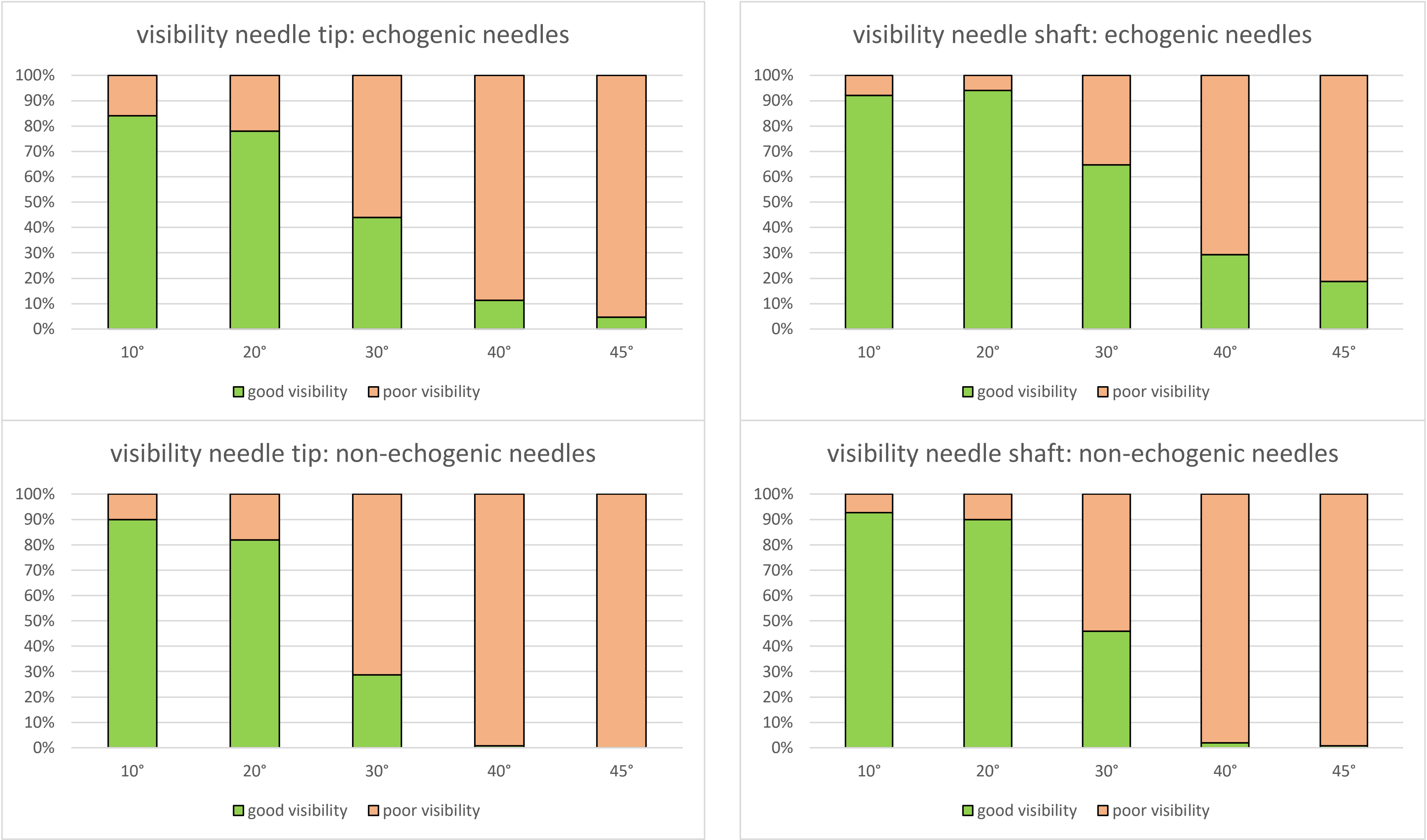

Supplemental Figure 1: Visibility of needle tip and needle shaft comparing echogenic needles with non-echogenic needles.
